# Lagging effects and prediction of pollutants and their interaction modifiers on influenza in northeastern China

**DOI:** 10.1186/s12889-023-16712-6

**Published:** 2023-09-19

**Authors:** Ye Chen, Weiming Hou, Weiyu Hou, Jing Dong

**Affiliations:** 1 Department of Infectious Disease, Shenyang Center for Disease Control and Prevention, 110100 Shenyang, Liaoning Province People’s Republic of China; 2 Shenyang Natural Focal Diseases Clinical Medical Research Center, 110100 Shenyang, Liaoning Province People’s Republic of China; 3 Department of Occupational and Environmental Health, School of Public Health, China Medical University, No.77 Puhe Road, 110122 Shenyang, People’s Republic of China; 4 The First Hospital of Shanxi Medical University, No.85 Jiefang South Road, 030012 Taiyuan, People’s Republic of China; 5 Key Laboratory of Environmental Stress and Chronic Disease Control & Prevention (China Medical University), Ministry of Education, No.77 Puhe Road, 110122 Shenyang, People’s Republic of China

**Keywords:** Pollutants, Influenza, NO_2_, Generalized additive model, Time series analysis

## Abstract

**Background:**

Previous studies have typically explored the daily lagged relations between influenza and meteorology, but few have explored seasonally the monthly lagged relationship, interaction and multiple prediction between influenza and pollution. Our specific objectives are to evaluate the lagged and interaction effects of pollution factors and construct models for estimating influenza incidence in a hierarchical manner.

**Methods:**

Our researchers collect influenza case data from 2005 to 2018 with meteorological and contaminative factors in Northeast China. We develop a generalized additive model with up to 6 months of maximum lag to analyze the impact of pollution factors on influenza cases and their interaction effects. We employ LASSO regression to identify the most significant environmental factors and conduct multiple complex regression analysis. In addition, quantile regression is taken to model the relation between influenza morbidity and specific percentiles (or quantiles) of meteorological factors.

**Results:**

The influenza epidemic in Northeast China has shown an upward trend year by year. The excessive incidence of influenza in Northeast China may be attributed to the suspected primary air pollutant, NO_2_, which has been observed to have overall low levels during January, March, and June. The Age 15–24 group shows an increase in the relative risk of influenza with an increase in PM_2.5_ concentration, with a lag of 0–6 months (ERR 1.08, 95% CI 0.10–2.07). In the quantitative analysis of the interaction model, PM_10_ at the level of 100–120 μg/m^3^, PM_2.5_ at the level of 60–80 μg/m^3^, and NO_2_ at the level of 60 μg/m^3^ or more have the greatest effect on the onset of influenza. The GPR model behaves better among prediction models.

**Conclusions:**

Exposure to the air pollutant NO_2_ is associated with an increased risk of influenza with a cumulative lag effect. Prioritizing winter and spring pollution monitoring and influenza prediction modeling should be our focus.

**Supplementary Information:**

The online version contains supplementary material available at 10.1186/s12889-023-16712-6.

## Introduction

Influenza is an acute viral respiratory illness in humans, usually characterized by fever, headache, muscle pain, weakness, nasal congestion, sore throat and cough. Seasonal epidemics of influenza viruses can spread rapidly and cause significant morbidity and mortality worldwide [1–3]. Globally, influenza is estimated to cause 3 to 5 million severe cases and 290,000 to 650,000 deaths related to respiratory infections every year [4]. While certain non-pharmaceutical interventions that can effectively control influenza in the early stages exist, such as the use of masks, hand washing, and other hygiene measures, or even closing schools, influenza is still a big threat to human health [5]. As evidenced by the trends of influenza incidence in such countries as Europe, the United States and Japan [6, 7], the situation remains grim and global preventive measures have little impact on the trend of influenza outbreaks. Currently few models can effectively predict influenza outbreaks. By monitoring influenza morbidity indicators on a daily basis after environmental factors that tend to predict outbreaks can be identified and modelled, we could predict influenza virus outbreaks in advance to reduce or even prevent influenza with associated costs.

In early studies we can assume that air pollution may contribute to influenza-induced morbidity. An epidemiological investigation suggests that particulate matter ≤ 10 μm (PM_10_) and ozone (O_3_) should be considered when forecasting the incidence of influenza [8, 9]. Influenza viruses have been detected in polluted waters, possibly originating from bird excretion carrying the virus, as per some studies [10]. As shown in the 2002–2003 SARS pandemic and the 2009 H_1_N_1_ influenza pandemic, influenza viruses are mainly transmitted through respiratory droplets. So air pollutants such as particulate matter (PM) and carbon monoxide (CO) may influence the transmission and prevalence of influenza viruses [11, 12]. In addition, secondary human-to-human transmission may occur and the outbreak may lead to the closure of schools and workplaces. Previous studies have investigated how meteorological factors facilitate the transmission of influenza among regions worldwide. A study in the UK showed that influenza viruses prefer low temperatures in temperate regions [13], while researchers in Canada have observed that the increase of influenza viruses is associated with low temperatures and high relative humidity [14]. The different subtypes of influenza viruses that have infected humans in recent decades include the H_10_N_8_, H_5_N_6_ and H_9_N_2_, most of which were firstly reported in China [15].

Numerous studies, both domestically and internationally, have investigated the correlation between the seasonal distribution patterns of influenza and meteorological and pollution factors. In China, Yuzhou Zhang et al. [16] explored the effect of different meteorological factors on influenza incidence in Shanghai by developing a distribution lagged nonlinear model (DLNM). Some researches indicated that in the multi-day lag model, there was a statistically significant correlation between SO_2_, NO_2_ and O_3_ concentrations and influenza risk between lags 0 and 1 [17]. Similarly, in Nanjing, Lei Huang et al. [18] found that PM_2.5_ and NO_2_ were associated with an increase in influenza cases. Air pollutants significantly affect the susceptibility of human respiratory epithelial cells to influenza virus infection by increasing virus attachment and entry.

The overall objective of this study is to explore influenza epidemic characteristics, lagged effects of pollutants and develop models suitable for predicting influenza virus outbreaks. Our specific objectives are to: a) screen environmental predictors of influenza outbreaks; b) evaluate the lagged and interaction effects of pollution factors; c) construct models for estimating influenza incidence in a hierarchical manner, selecting appropriate models for different characteristics.

## Materials and methods

**Fig. 1:**
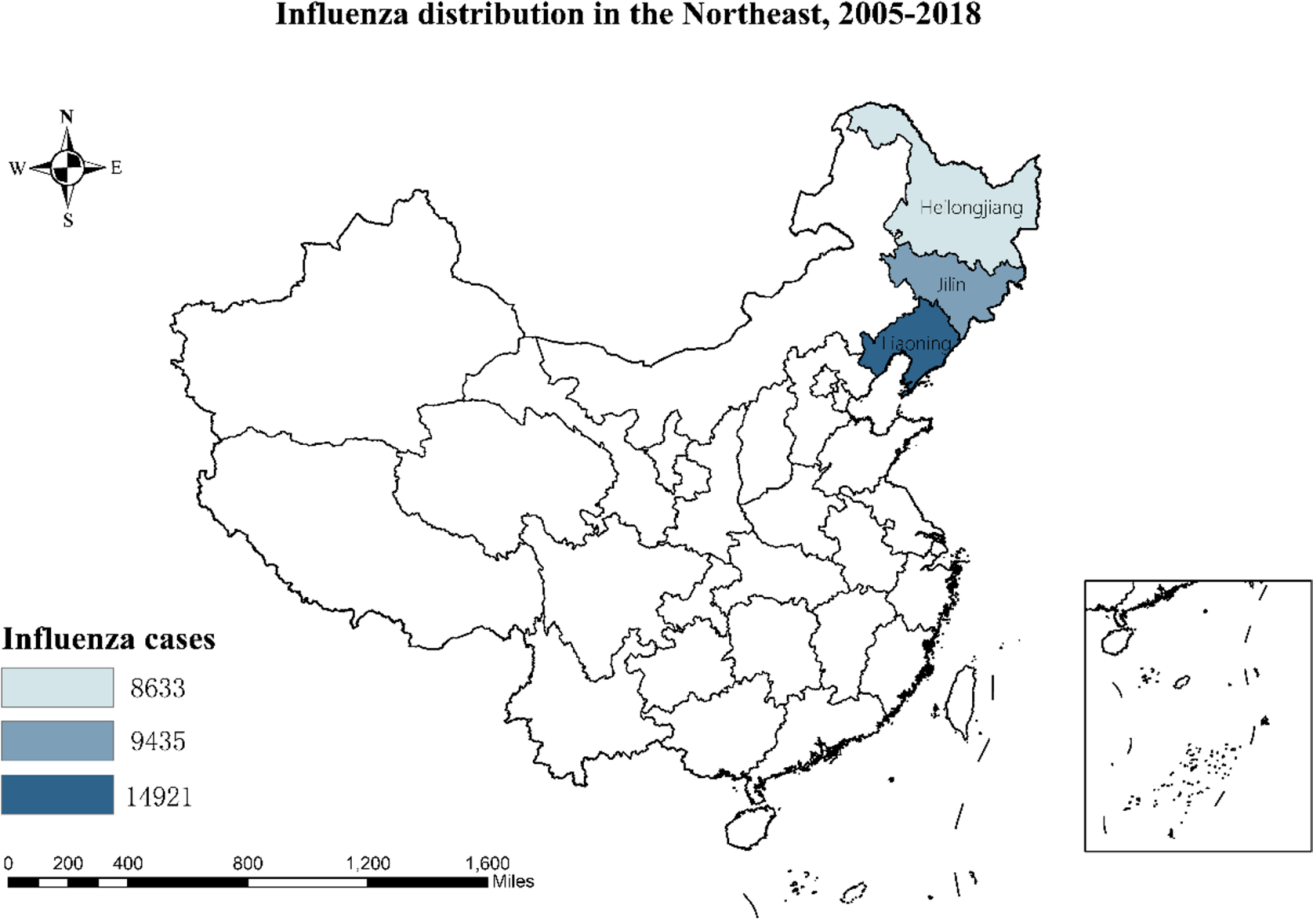
The geographical location of Northeast in China The map was created by ArcGIS 10.3 (Environmental Systems Research Institute; Redlands, CA, USA). The base map was acquired from the data center for geographic sciences and natural sources research, CAS (http://www.resdc.cn/data.aspx?DATAID=201)

Figure 1 shows the geographical location of the study area—Heilongjiang, Jilin and Liaoning provinces, which lie between 120° and 135° E and 40° and 53° N latitude in China. These three provinces are located in Northeast China with a medium level of economic development and population density.

We collected influenza case surveillance data from the National Public Health Data Centre of China between 2005 and 2018. All patients are diagnosed according to the criteria of influenza management issued by the Ministry of Health of the People's Republic of China. We obtained the corresponding daily weather data including air temperature, dew point temperature etc. from the China Meteorological Data Sharing Service. Pollutant information is originally from the National Oceanic and Atmospheric Administration (NOAA) including CO, NO_2_, O_3_ etc.

## Statistical methods

To address missing values in the influenza epidemic and meteorological pollution data, we use multiple interpolation to fill them. LASSO regression analysis is used for feature selection in response to the effects of meteorological and pollution factors. We develop quantile regression models and generalized additive models [13] with a maximum lag of 6 months to assess the extreme effects of pollution and meteorological factors on influenza cases, lags, and interactions between pollutants, respectively. Finally, we make prediction by complex regression models. All analysis in our study is performed in R software (version 4.1.3).

### The screening of lasso regression with environmental variables

There are N sets of observations, each consisting of a total response variable *y* and *p* associated characteristic variables *x*_*i*_ = (*x*_*i1*_, …, *x*_*ip*_) ^*T*^. A linear regression model can be set as follows:

$${y}_{i}= {\beta }_{0} + \sum_{j=1}^{p}{x}_{ij}{\beta }_{j} + {e}_{i}, i=1, \cdots , N$$

where* β*_*0*_ and *β* = (*β*_*1*_, *β*_*2*_, …, *β*_*p*_) are unknown parameters and *e*_*i*_ is the error term. The introduction of some variables in practical problems not only complicates the calculation, but also risks increasing the data covariance, thus affecting the model fit. We can use lasso regression to estimate the parameters by solving the following problem:

$${\mathrm{min}}_{\beta 0,\beta }\sum_{i=1}^{N}{\left({y}_{i}- {\beta }_{0}- \sum_{j=1}^{p}{x}_{ij}{\beta }_{j}\right)}^{2}, \parallel \beta {\parallel }_{1}\le t$$

where $\parallel\beta\parallel_1=\sum_{j=1}^p\left|\beta_j\right|$ is the ${l}_{1}$ parametrization of $\beta$ and *t* is the specified tuning parameter. Overall, the lasso method improves the overall prediction accuracy, and the inclusion of the constraint term compresses the coefficients of some of the eigenvariables in the model to zero, thus enabling the selection of important variables among the many eigenvariables.

### *The lagging and interaction effect of generalized additive model *[13]

The models are listed as follows (Model 1):

$$log\left[E\left({Y}_{t}\right)\right]= \alpha 1+ \sum S\left(M,df,lag\right)+S\left(\mathrm{Time},df\right)+F\mathrm{actor}\left({\mathrm{Month}}_{t}\right)+{\beta Year}_{t}$$

Here, *Y*_*t*_ is the number of monthly counts of influenza cases in monthly t; *α1* is the intercept of the whole model; *S* () is a smoothing function, and the penalty spline method is often used to smooth the parameters; *M* represents the estimated environmental variable related to influenza; *β* is the regression coefficients. The optimal degrees of freedom (*df*) for the spline function are estimated by Akaike information criterion for Poisson (AIC) and Minimum partial regression coefficient (PACF_min_) criteria.

Subsequently, we explore the interaction of pollutants on the prevalence of influenza. The model can be written as follows (Model 2):

$$log\left[E\left(Yt\right)\right]= \alpha 2+ S\left({X}_{1},{X}_{2},\mathrm{lag},df\right)+S\left({\mathrm{X}}_{3},\mathrm{lag},df\right)+\sum S\left(\mathrm{M},df\right)+S\left(\mathrm{Time}, df\right)$$

*α*2 is the intercept; *X*_1_ indicates two of the interaction factors whereas *X*_3_ denote the other one; *S*(*X*_1_, *X*_2_) is a spline function of the interaction between the parameters *X*_1_ and *X*_2_*.* M is the meteorological factors.

### The establishment of Gradient boosting regression tree (GBRT) and Gaussian process regression (GPR) model

If the input training set is: T = {(× 1, y1), (× 2, y2), …, (xN, yN)}, the training samples i = 1, 2, …, N, the number of iteration rounds t = 1, 2, …, T, and the loss function is L, then the GBRT algorithm is divided into the following three steps:

First, initialize the weak learner: $f_0\left(x\right)=\mathrm{argmin}\;\sum\limits_{i=1}^N\;L\left(y_i,\;c\right)$  

Next, calculate the negative gradient r_ti_ and the output of each leaf node region of the regression tree, R_tm_ output value c_tm_, and update the strong learner:

$${\mathrm{r}}_{\mathrm{ti}}= -{\left[\frac{\partial \mathrm{L}\left({\mathrm{y}}_{\mathrm{i}},\mathrm{ f}\left({\mathrm{x}}_{1}\right)\right)}{\partial \mathrm{f}}\right]}_{\mathrm{f}\left(\mathrm{x}\right)={\mathrm{f}}_{\mathrm{t}-1}\left(\mathrm{x}\right)}$$

$${\mathrm{c}}_{\mathrm{tm}}=\mathrm{argmin }\sum_{{\mathrm{X}}_{\mathrm{i}}\upepsilon {\mathrm{R}}_{\mathrm{tm}}}\mathrm{L}\left({\mathrm{y}}_{\mathrm{i}}, {\mathrm{f}}_{\mathrm{t}-1}\left({\mathrm{X}}_{\mathrm{i}}\right),\mathrm{ c}\right)$$

$${\mathrm{f}}_{\mathrm{t}}\left(\mathrm{x}\right)= {\mathrm{f}}_{\mathrm{t}-1}\left(\mathrm{x}\right)+ \sum_{\mathrm{m}=1}^{\mathrm{M}}{\mathrm{c}}_{\mathrm{tm}}\mathrm{I}\left({\mathrm{X \epsilon R}}_{\mathrm{tm}}\right)$$

Finally, get strong learners: 

$$f\;\left(x\right)=\;f_T\;\left(x\right)\;=f_0\left(x\right)+\sum_{t=1}^T\sum_{m=1}^M\;c_{tm}I\left(x\mathit\in R_{tm}\right)$$

From a function space perspective, a Gaussian process [19] is defined to describe the distribution of the function (f(x)). The GP is the set of any finite number of random variables that have a joint Gaussian distribution, and its properties are determined entirely by the mean and covariance functions, that is:

$$\left\{\begin{array}{l}\mathrm{m}\left(\mathrm{x}\right)=\mathrm{E}\left[\mathrm{f}\left(\mathrm{x}\right)\right]\\ \mathrm{k}\left(\mathrm{x}, {\mathrm{x}}^{\mathrm{^{\prime}}}\right)=\mathrm{E}\left[\left(\mathrm{f}\left(\mathrm{x}\right)-\mathrm{m}\left(\mathrm{x}\right)\right)\left(\mathrm{f}\left({\mathrm{x}}^{\mathrm{^{\prime}}}\right)-\mathrm{m}\left({\mathrm{x}}^{\mathrm{^{\prime}}}\right)\right)\right]\end{array}\right.$$

where x,x' ∈ R are arbitrary random variables. Thus GP can be defined as f (x) ~ GP(m(x), k(x, x')) and the mean function is generally taken to be 0 (m(x) = 0). For the regression model as follows:

$$y=f\left(x\right)+\varepsilon$$

where *x* is the input value, *f* is the function value, and *y* is the observation plus the observation affected by noise, if noise $\varepsilon \sim \mathrm{N}\left(0,{\upsigma }_{\mathrm{n}}^{2}\right)$ yields a priori distribution of the observation *y* as follows:

$$\mathrm{y }\sim \mathrm{ N}\left(0,\mathrm{K}\left(\mathrm{X},\mathrm{X}\right)+{\upsigma }_{\mathrm{n}}^{2}{\mathrm{I}}_{\mathrm{n}}\right)$$

## Results

### Influenza surveillance in Northeast China

From 2005 to 2018, a total of 32,989 influenza cases were reported in the three eastern provinces of China, showing an increasing trend every year (Table 1 and Fig. 1). Heilongjiang province exhibited a significantly high level of epidemic in the first few years, followed by Liaoning province, which has consistently been the main epidemic area since then and had 14,921 reported cases of influenza by 2018. Young children aged 5–14 years and young adults aged 25–59 years had the highest incidence of influenza, accounting for 55.35% of all reported cases (Table 1). Significant differences in the incidence of influenza were observed in terms of seasonality, age, and region (*P* < 0.05).

**Table 1:** Distribution of the influenza cases by age, region and season group in northeast China, 2005–2018

Characteristic	0–4	5–14	15–24	25–59	≥ 60	Total	Population	ILI
	No of ILI cases (%)						(10^4^)	(10^–2^%)
Year								
2005	15(9.32%)	74(45.96%)	13(8.07%)	46(28.57%)	13(8.08%)	161	10757	0.01
2006	37(22.70%)	73(44.79%)	22(13.50%)	29(17.79%)	2(1.23%)	163	10917	0.01
2007	132(37.93)	141(40.52%)	26(7.47%)	42(12.07%)	7(2.01%)	348	10952	0.03
2008	119(40.34%)	100(33.9%)	22(7.46%)	48(16.27%)	6(2.03%)	295	10874	0.03
2009	324(12.83%)	889(35.21%)	684(27.09%)	559(22.14%)	69(2.73%)	2525	10907	0.23
2010	677(27.15%)	681(27.31%)	359(14.39%)	668(26.78%)	109(4.37%)	2494	10955	0.23
2011	178(22.97%)	155(20.00%)	57(7.35%)	322(41.55%)	63(8.13%)	775	10966	0.07
2012	438(19.57%)	477(21.31%)	215(9.61%)	906(40.48%)	202(9.03%)	2238	10973	0.20
2013	436(21.26%)	332(16.19%)	165(8.04%)	874(42.61%)	244(11.90%)	2051	10976	0.19
2014	782(21.66%)	1115(30.89%)	307(8.50%)	1066(29.53%)	340(9.42%)	3610	10976	0.33
2015	666(25.87%)	463(17.99%)	174(6.76%)	916(35.59%)	355(13.79%)	2574	10947	0.24
2016	963(24.34%)	1080(27.30%)	270(6.83%)	1200(30.33%)	443(11.20%)	3956	10910	0.36
2017	1070(24.62%)	1173(26.99%)	368(8.47%)	1196(27.52%)	539(12.40%)	4346	10875	0.40
2018	2181(29.26%)	1653(22.18%)	381(5.11%)	1982(26.59%)	1256(16.85%)	7453	10836	0.69
Province Region								
Heilongjiang	1973(22.85%)	2915(33.77%)	1172(13.58%)	2025(23.46%)	548(6.35%)	8633	3773	2.29
Jilin	2306(24.44%)	2316(24.55%)	947(10.04%)	2930(31.05%)	936(9.92%)	9435	2704	3.49
Liaoning	3739(25.06%)	3175(21.28%)	944(6.33%)	4899(32.83%)	2164(14.50%)	14921	4359	3.42
Total	8018(24.31%)	8406(25.48%)	3063(9.28%)	9854(29.87%)	3648(11.06%)	32989	10836	3.04
Seasons								
Spring(Mar-May)	1968(23.67%)	2410(28.99%)	675(8.12%)	2310(27.79%)	950(11.43%)	8313		
Summer(Jun-Aug)	426(19.00%)	283(12.62%)	175(7.81%)	896(39.96%)	462(20.61%)	2242		
Autumn(Sep-Nov)	935(16.22%)	1420(24.64%)	772(13.40%)	1908(33.11%)	728(12.63%)	5763		
Winter(Dec-Feb)	4689(28.13%)	4293(25.75%)	1441(8.64%)	4740(28.43%)	1508(9.05%)	16671		

### The screening and extreme effect for meteorological and pollutant factors among influenza prevalence

**Fig. 2:**
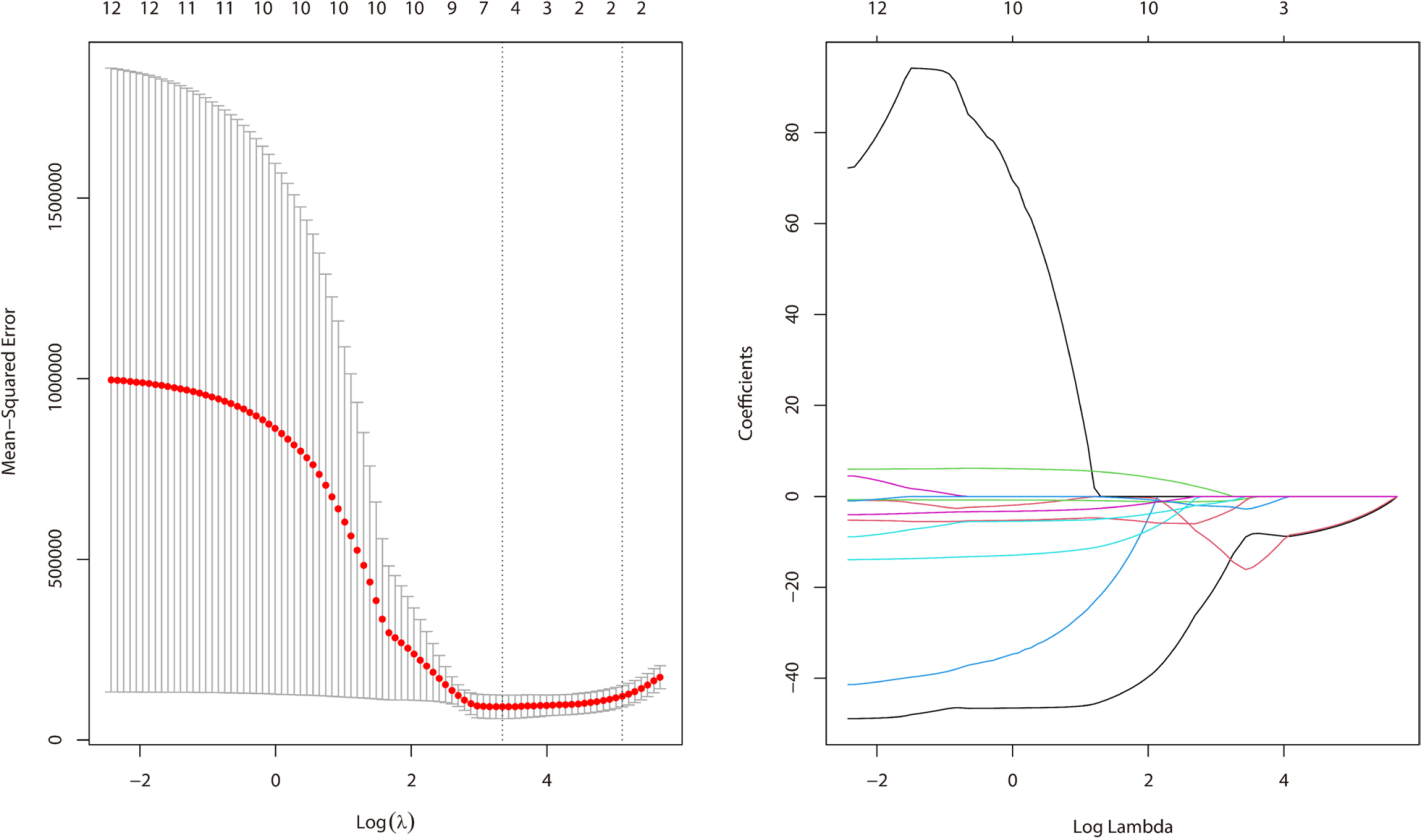
The process of lasso regression variable screening

We apply a fivefold cross-validation to select a model with small and stable error fluctuations and a parameter λ of 28.3327. The results of the runs are shown in Fig. 2 and Table S1. After the initial selection of the lasso method, six of the variable variables including air temperature (AT), dew point temperature (DPT), sea level pressure (SLP), NO_2_, PM_10_ and PM_2.5_ are selected and the coefficients of the other independent variables are contracted to zero.

### Exposure–response relationships for pollutants with different lag times

**Fig. 3:**
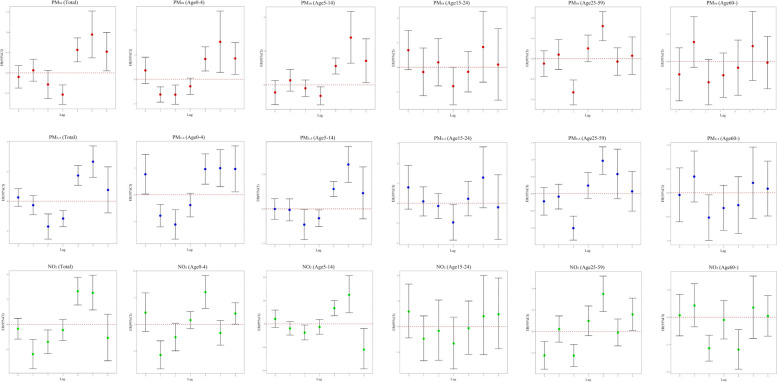
The associations between ambient air pollution and monthly Influenza prevalence with total and all ages

From the line graph of Figure S1 regression coefficients, we can see that SLP does not fluctuate at different levels, while PM_2.5_ fluctuates at different concentrations, but the overall effect increases with the quantile. In addition, PM_10_, NO_2_ and AT has a negative effect on influenza incidence at different levels, and the overall effect also increases with the quantile. Significant differences were observed between the age groups of 25–59 years and 60 + year. In Table 2 and Fig. 3, in the single-pollutant model we find a negative association between short-term exposure to NO_2_ (within 1 month) and monthly influenza incidence (ERR-2.68% (-4.72%, -0.60%)), and this implies that low levels of NO_2_ may be the most responsible air pollutant for excess influenza incidence. The Age 25–59 years group is the most susceptible to NO_2_, followed by the Age 0–4 years group, and the ERR increases with lag in both groups, with essentially no lag in the 60 + years age group. NO_2_ showed a positive correlation with influenza incidence in patients aged 15–24 and 25–59 years at a 3-month lag. At a 5-month lag, there was a positive correlation between influenza incidence and NO_2_ in patients aged 0–4, 15–24, and 25–59 years. At a maximum lag time of 6 months, the influenza incidence in patients aged 15–24 shows a positive correlation with PM_2.5_.

**Table 2:** Cumulative ERR of each pollutant at different lags (Unit: %)

Lags (Month)	Pollutants	Total		Age0-4		Age5-14		Age15-24		Age25-59		Age60-	
		ERR(95%CI)	Sig	ERR(95%CI)	Sig	ERR(95%CI)	Sig	ERR(95%CI)	Sig	ERR(95%CI)	Sig	ERR(95%CI)	Sig
**Lag0-1**	PM_10_	0.17(-0.16,0.50)	0.308	0.19(-0.65,1.03)	0.665	0.25(-0.53,1.03)	0.536	0.58(-0.70,1.88)	0.378	-0.07(-0.51,0.36)	0.740	0.26(-0.35,0.88)	0.403
	PM_2.5_	0.17(-0.21,0.55)	0.387	-0.58(-1.30,0.15)	0.117	0.26(-0.66,1.19)	0.584	0.59(-0.60,1.80)	0.330	-0.22(-0.74,0.31)	0.416	0.37(-0.33,1.08)	0.301
	NO_2_	**-2.68(-4.72,-0.60)**	0.012*	**-5.17(-8.56,-1.66)**	0.004**	-1.46(-6.16,3.48)	0.556	-0.58(-6.63,5.87)	0.857	-0.58(-6.63,5.87)	0.857	1.56(-1.29,4.50)	0.285
**Lag0-2**	PM_10_	0.08(-0.38,0.53)	0.744	-0.75(-1.50,0.00)	0.051	0.34(-0.73,1.42)	0.535	0.69(-0.99,2.40)	0.424	-0.57(-1.16,0.02)	0.059	0.12(-0.79,1.05)	0.791
	PM_2.5_	0.01(-0.46,0.48)	0.970	-0.27(-1.26,0.73)	0.595	0.27(-0.90,1.46)	0.653	0.25(-1.47,2.00)	0.774	**-0.81(-1.38,-0.23)**	0.006**	0.21(-0.70,1.14)	0.648
	NO_2_	-0.80(-2.58,1.01)	0.384	-3.02(-6.93,1.06)	0.144	0.11(-4.59,5.05)	0.964	-2.38(-8.38,4.00)	0.455	-2.38(-8.38,4.00)	0.455	0.68(-2.62,4.10)	0.689
**Lag0-3**	PM_10_	**-0.91(-1.50,-0.32)**	0.002**	-0.38(-1.47,0.73)	0.503	-0.62(-2.06,0.85)	0.407	0.66(-1.26,2.61)	0.504	**-1.47(-2.34,-0.59)**	0.001**	-0.98(-2.09,0.14)	0.086
	PM_2.5_	**-0.67(-1.19,-0.15)**	0.012*	-0.40(-1.48,0.69)	0.472	-0.33(-1.49,0.83)	0.572	0.17(-1.71,2.08)	0.862	**-1.19(-1.98,-0.39)**	0.004**	-0.67(-1.66,0.34)	0.193
	NO_2_	**-1.92(-3.56,-0.26)**	0.024*	2.19(-0.38,4.84)	0.096	-0.50(-4.68,3.87)	0.821	**3.19(0.11,6.37)**	0.042*	**3.19(0.11,6.37)**	0.042*	-1.70(-4.86,1.57)	0.305
**Lag0-4**	PM_10_	**-0.53(-1.05,0.02)**	0.042*	0.09(-1.07,1.26)	0.883	-0.45(-1.73,0.83)	0.487	0.48(-1.53,2.53)	0.640	-0.72(-1.56,0.13)	0.099	-0.84(-1.97,0.29)	0.145
	PM_2.5_	**-0.53(-1.03,-0.02)**	0.043*	-0.06(-1.27,1.17)	0.928	-0.30(-1.52,0.93)	0.633	0.03(-2.05,2.16)	0.997	-0.74(-1.55,0.08)	0.076	-0.66(-1.70,0.39)	0.218
	NO_2_	-1.62(-3.36,0.15)	0.072	-0.31(-4.76,4.35)	0.893	-0.52(-4.64,3.79)	0.811	2.79(-0.14,5.80)	0.062	2.79(-0.14,5.80)	0.062	-1.48(-4.92,2.09)	0.412
**Lag0-5**	PM_10_	-0.41(-0.96,0.15)	0.151	0.28(-0.85,1.43)	0.625	-1.29(-2.73,0.17)	0.084	0.79(-1.20,2.83)	0.439	-0.23(-0.99,0.54)	0.556	-0.52(-1.55,0.52)	0.324
	PM_2.5_	-0.26(-0.87,0.35)	0.405	0.51(-0.82,1.85)	0.456	-1.08(-2.71,0.58)	0.201	0.76(-1.60,3.17)	0.532	0.03(-0.71,0.77)	0.937	-0.54(-1.63,0.56)	0.332
	NO_2_	-0.40(-2.65,1.89)	0.728	**3.12(0.22,6.10)**	0.035*	-3.95(-9.89,2.38)	0.215	**2.79(0.03,5.63)**	0.048*	**2.79(0.03,5.63)**	0.048*	-1.01(-4.44,2.55)	0.574
**Lag0-6**	PM_10_	**-1.44(-2.06,-0.82)**	0.000**	**-1.50(-2.39,-0.61)**	0.001**	**-1.98(-3.45,-0.48)**	0.010**	0.34(-1.63,2.35)	0.739	-0.46(-1.16,0.25)	0.205	-0.86(-1.92,0.21)	0.116
	PM_2.5_	**-2.02(-2.94,1.08)**	0.000**	-1.50(-3.20,0.22)	0.087	**-3.01(-5.02,-0.95)**	0.004**	**1.08(0.10,2.07)**	0.031*	-0.35(-1.23,0.53)	0.434	-0.65(-1.94,0.65)	0.327
	NO_2_	**-9.67(-13.28,-5.90)**	0.000**	**-8.26(-14.42,-1.65)**	0.015*	-15.81(-23.75,-7.04)	0.001**	-10.51(-24.34,5.85)	0.195	-10.51(-24.34,5.85)	0.195	-1.82(-5.90,2.42)	0.394

### Interaction and comparison of multiple-pollutant model

**Fig. 4:**
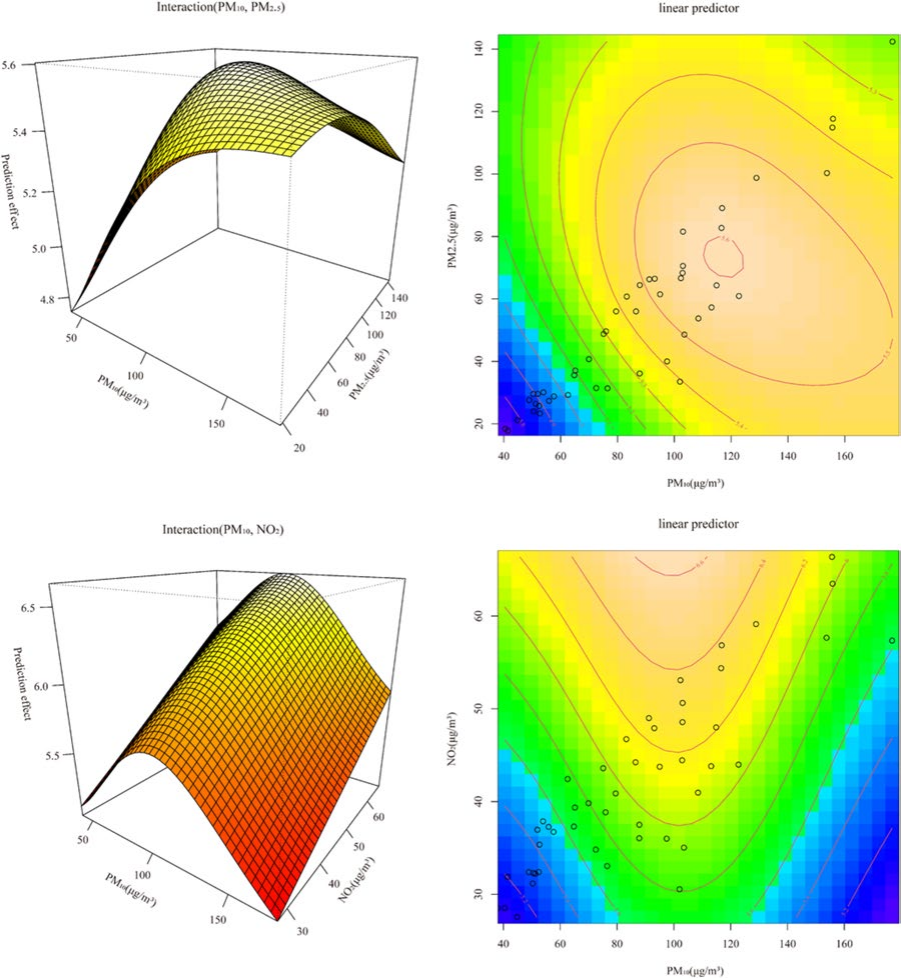
The fitting interactions of the association among pollutants and Influenza cases based on the generalized additive model

We develop a multi-pollutant model with a single-day lagged ERR maximum and significance test, yielding a lagged day of 5 days for PM_10_, PM_2.5_ and 4 days for NO_2_. Figure S2 revealed that PM_10_ has interactive effects with PM_2.5_ and NO_2_ on influenza incidence. Additionally, there is a weak positive correlation between pollutants and the risk of incidence. Moreover, ambient temperature (AT) exhibits positive correlation with the risk of incidence at low temperatures and inverse correlation at high temperatures, while dew point temperature (DPT) shows an inverse trend compared to AT. Figure 4 indicated a non-linear effect of pollutants on influenza onset, with PM_10_ levels of 100–120 μg/m^3^ and PM_2.5_ levels of 60–80 μg/m^3^, and NO_2_ levels above 60 μg/m^3^ exhibiting the greatest impact. Results from the statistical test presented in Table 3 suggest that the PM_10_ and PM_2.5_ interaction model is better (R^2^ = 99.1%). These findings demonstrate the significant impact of air pollutants on influenza onset.

**Table 3:** Test of interaction model of multiple pollution factors

Interaction	Parameters	edf	Ref.df	χ^2^	*P*	R^2^(adj)	Deviance explained	UBRE
s(PM_10_lag5,PM_2.5_lag5)	PM_10_lag5,PM_2.5_lag5	2.899	2.978	58.74	< 2e-16***	0.991	99.50%	2.326
	NO_2_lag4	1.742	1.894	20.23	2.82e-05***			
	time	10.549	10.83	607.2	< 2e-16***			
	AT	6.493	6.866	266.9	< 2e-16***			
	DPT	6.908	6.979	283.3	< 2e-16***			
	SLP	1.966	2.293	27.3	1.04e-05***			
s(PM_10_lag5,NO_2_lag4)	PM_10_lag5,NO_2_lag4	2.962	2.994	106.2	< 2e-16***	0.989	99.50%	2.527
	PM_2.5_lag5	1.002	1.003	10.22	0.00142**			
	time	10.835	10.97	737.2	< 2e-16***			
	AT	6.775	6.951	229.1	< 2e-16***			
	DPT	6.586	6.901	268.7	< 2e-16***			
	SLP	2.07	2.411	26.47	1.22e-05***			

From the comparison of the parameters of the two modelling approaches in Table 4, the model fit is the best in Liaoning Province among different regions (R^2^ > 70%), and the model fit is the best among Age 25–59 groups, while the GPR model shows the same fit as the GBRT model.

**Table 4:** Comparison of the prediction results with the gradient boosted regression tree (GBRT) and gaussian distribution regression (GPR) models

Model	Stratification	Parameters	cv.fold	Training set			Test set		
				RMSE	R^2^	MAE	RMSE	R^2^	MAE
GBRT	Total	ntree = 301, shrinkage = 0.01	5	172.93	0.68	101.93	545.77	0.77	347.27
	Heilongjiang	ntree = 170, shrinkage = 0.01	5	101.96	0.43	52.91	162.11	0.72	103.09
	Jilin	ntree = 414, shrinkage = 0.01	5	50.26	0.72	33.67	240.82	0.72	171.42
	Liaoning	ntree = 560, shrinkage = 0.01	5	46.38	0.79	27.38	155.14	0.78	85.29
	Age0-4	ntree = 369, shrinkage = 0.01	5	47.63	0.70	31.45	190.33	0.77	114.36
	Age5-14	ntree = 162, shrinkage = 0.01	5	100.67	0.42	47.90	150.98	0.62	75.65
	Age15-24	ntree = 267, shrinkage = 0.01	5	23.39	0.50	11.60	24.08	0.73	15.40
	Age25-59	ntree = 445, shrinkage = 0.01	5	27.23	0.81	18.64	129.02	0.77	85.24
	Age60-	ntree = 344, shrinkage = 0.01	5	9.99	0.67	6.39	86.49	0.73	68.67
GPR	Total	sigma = 0.3410, tol = 0.0005, error = 0.32	5	170.56	0.70	101.35	540.63	0.82	332.61
	Heilongjiang	sigma = 0.3410, tol = 0.0005, error = 0.49	5	90.71	0.54	48.61	143.50	0.79	91.62
	Jilin	sigma = 0.3410, tol = 0.0005, error = 0.35	5	55.72	0.68	37.30	243.98	0.81	163.64
	Liaoning	sigma = 0.3410, tol = 0.0005, error = 0.19	5	44.61	0.84	31.41	169.93	0.76	104.32
	Age0-4	sigma = 0.3410, tol = 0.0005, error = 0.33	5	49.23	0.70	33.39	195.84	0.80	119.73
	Age5-14	sigma = 0.3410, tol = 0.0005, error = 0.47	5	88.21	0.57	44.56	140.75	0.65	78.62
	Age15-24	sigma = 0.3410, tol = 0.0005, error = 0.44	5	21.34	0.61	11.46	18.38	0.81	13.28
	Age25-59	sigma = 0.3410, tol = 0.0005, error = 0.22	5	29.58	0.80	21.00	129.21	0.82	83.95
	Age60-	sigma = 0.3410, tol = 0.0005, error = 0.28	5	9.33	0.74	6.71	88.05	0.74	68.70

## Discussion

Based on this study, it is determined that influenza epidemics in the northeastern region of China exhibit pronounced seasonality of winter-spring and demonstrate an upward trend annually. Prior to 2018, the province of Liaoning continued to be the primary epicenter of influenza outbreaks within the northeastern region, with a higher prevalence of cases observed among young children and adolescents, which could be attributed to their relatively weaker immune systems rendering them more susceptible to influenza viral infections [20].

Our single-pollutant lagged modeling yields intriguing results, indicating that lower concentrations of NO_2_ in January, March, and June may be the primary contributing factor to excessive influenza incidence. Exposure to NO_2_ can lead to reduced virus-specific immunity and increased cellular inflammation, potentially causing the onset of influenza virus, regardless of whether it occurs before or after respiratory virus infection. The relative risk of influenza associated with NO_2_ exposure increased with higher NO_2_ concentrations in the age groups of 0–4, 15–24, and 25–59. This suggests that young adults may have a higher susceptibility to influenza under NO_2_ exposure due to the rapid release of immune cells stimulated by the virus, resulting in an increased relative release of immune cells. The disruption of immune cell homeostasis caused by the rapid release of immune cells stimulated by influenza viruses may explain the higher susceptibility of young adults to influenza under NO_2_ exposure. This could be the result of a relative increase in immune cell release in this age group [21]. In the age group of 15–24, the relative risk of influenza associated with PM_2.5_ exposure increased with higher PM_2.5_ concentrations at lag 0–6 months. This suggests that long-term exposure to high levels of PM_2.5_ beyond 6 months may increase the risk of influenza. This was similar to the results of a monitoring study that found: Age 0–4 were significantly susceptible to PM_10_ and NO_2_; Age 5–14 were significantly susceptible to PM_2.5_ and PM_10_; and Age 15–24 were significantly susceptible to all air pollutants analyzed [18]. During the remaining lag months with low concentrations of PM_2.5_, PM_10_ and NO_2_, the onset of influenza is mainly attributed to factors other than air pollution, as indicated by ERR < 0. This may be due to low levels of external pollution, which increase population activity during high influenza season (spring, winter) and result in aggregated activity, thereby enhancing the risk of influenza.

The longitudinal study shows that the highest overall risk (ERR) of influenza onset is observed for pollutants with a lag of 5–6 months, indicating that long-term exposure to pollutants may primarily promote influenza onset. However, in the age groups of 15–24 and 60 + , NO_2_ exposure is associated with a higher risk of influenza onset at an early stage. The lagged effects of PM_2.5_, PM_10_ and NO_2_ are characterized by a bimodal distribution, with a significant decrease in the risk of influenza onset during the first 2 months of exposure. However, the risk of influenza onset is found to be higher during the 2nd-3rd and 4th-5th months of exposure to these pollutants. Several studies have suggested that influenza viruses exhibit higher sensitivity and pathogenicity during the winter season, based on experimental findings [22]. This might also be consistent with the findings of some surveys: The correlation between air pollutants and influenza varies by season and region, with higher effects estimated for the cold season, eastern and central regions, and provinces with wetter conditions and larger populations [23]. In the multi-pollutant lagged interaction model, ambient temperature (AT) is found to be positively correlated with the risk of influenza onset at low temperatures, while pollutants are only weakly positively correlated with the risk of morbidity. This suggests that lower temperatures may facilitate the spread of pollutants, thereby exacerbating the spread of influenza viruses and causing the onset of influenza. These findings are consistent with the seasonal characteristics of influenza onset, which predominantly occur during the winter and spring seasons in this study. The quantitative analysis of interaction models reveals that the interaction between PM_10_ and PM_2.5_ has a more significant effect on influenza onset. Several studies have suggested that particulate matter (PM) may stimulate macrophage apoptosis in lung tissue, which could exacerbate the damage caused by influenza viruses to the respiratory tract. This may explain why the combined effect of PM_10_ and PM_2.5_ is more detrimental to influenza onset [24].

During heavy pollution, reduced outdoor activities and increased indoor activities can heighten the risk of influenza [25]. Hence, the significance of the current study lies in the investigation of the link between pollutants and the development of influenza, which is in line with various domestic and international studies [17, 26, 27].

Temperature and sea level pressure are the most relevant meteorological factors in our study, as they can affect the transmission of pathogens and impact human immune function, leading to respiratory disease development [28–30]. In our final GAM model, we did not find a significant association between meteorological factors and influenza incidence. This may be due to the potential confounding effect of regional environment and lifestyle habits on the transmission of influenza.

Our analysis involves multiple models exploring the relationship between environmental factors and influenza incidence, and subsequent subgroup analyses demonstrate significant differences in the predictions made by region and age group. Interestingly, the Gaussian process regression (GPR) model outperforms the Gradient Boosting Regression Tree (GBRT) model in terms of predictive accuracy. In conclusion, the study suggests that Liaoning Province is proficient in predicting influenza outbreaks and accounting for environmental factors. Moreover, it serves as a suitable representative region for the northeastern part of China. Our study also finds that the model's fit and validation are satisfactory for the age group of 25–59 years, who are susceptible to influenza outbreaks. However, the predictive stability is suboptimal for the age group of 5–14 years, possibly due to the clustering of young individuals during the cold season.

It is notable that our study quantifies the impact of various factors on influenza incidence. The use of the GAM model allows us to control important confounding factors and examine the long-term monthly lagged effects of co-exposure to PM_10_ and PM_2.5_. However, it should be noted that our study is conducted at an aggregate level and does not involve individual-level analysis. And the data is only collected from the Northeast region, so caution is needed when extending our findings to other regions. However, our study provides a starting point for future population epidemiology studies with larger samples and broader geographic coverage.

### Supplementary Information

**Additional file 1.**

## Data Availability

The datasets analyzed during the current study are available in the National Public Health Data Centre of China (https://www.phsciencedata.cn/), the China Meteorological Data Sharing Service (www.data.cma.cn) and the National Oceanic and Atmospheric Administration (NOAA**)**.
