# Supplementary material for: Lagging effects and prediction of pollutants and their interaction modifiers on influenza in northeastern China
Source: BMC Public Health. 2023 Sep 19;23:1826. doi: 10.1186/s12889-023-16712-6 (PMC10510220; doi:10.1186/s12889-023-16712-6)
Supplement: Supplementary file 1 — Additional file 1. [file 12889_2023_16712_MOESM1_ESM.doc]

**Supplementary material**

**Lagging effects and prediction of pollutants and their interaction modifiers on influenza in northeastern China**

**Contents**

**1.Figure S1** Trend chart and confidence interval (grey area) of estimated coefficients of meteorological and pollution factors under different quantiles (horizontal axis: 0.25-0.75)…………………………………………………………………………….S2

**2.Figure S2** Comparison of interaction exposure-response curves of Pollutants-Influenza prevalence……………………………………………………...S3

**3.Table S1** The estimated coefficients for least absolute shrinkage and selection operator (LASSO) regression among factors with influenza………………………..S4

**Figure S1** Trend chart and confidence interval (grey area) of estimated coefficients of meteorological and pollution factors under different quantiles (horizontal axis: 0.25-0.75).


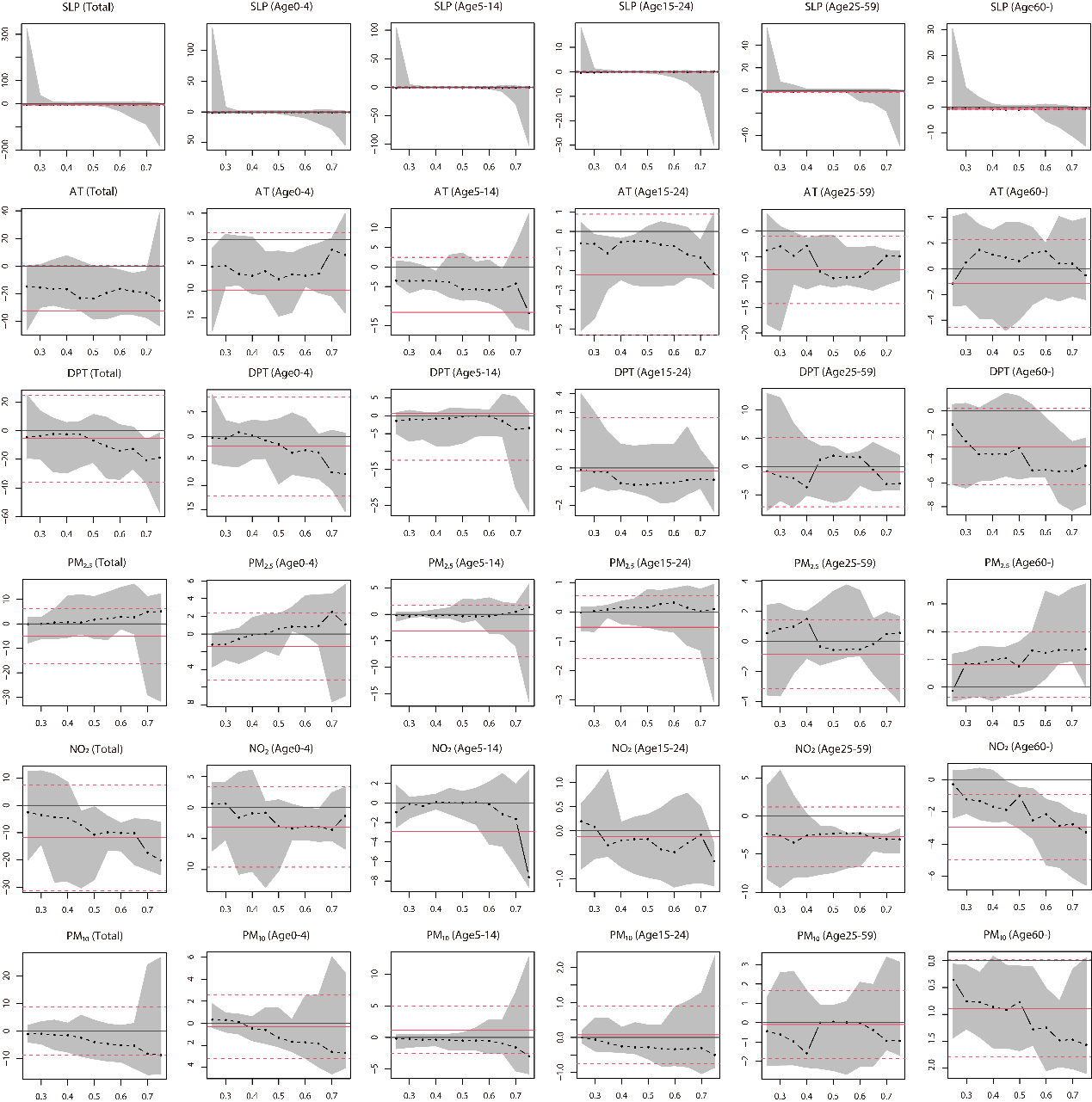


**Figure S2** Comparison of interaction exposure-response curves of Pollutants-Influenza prevalence.


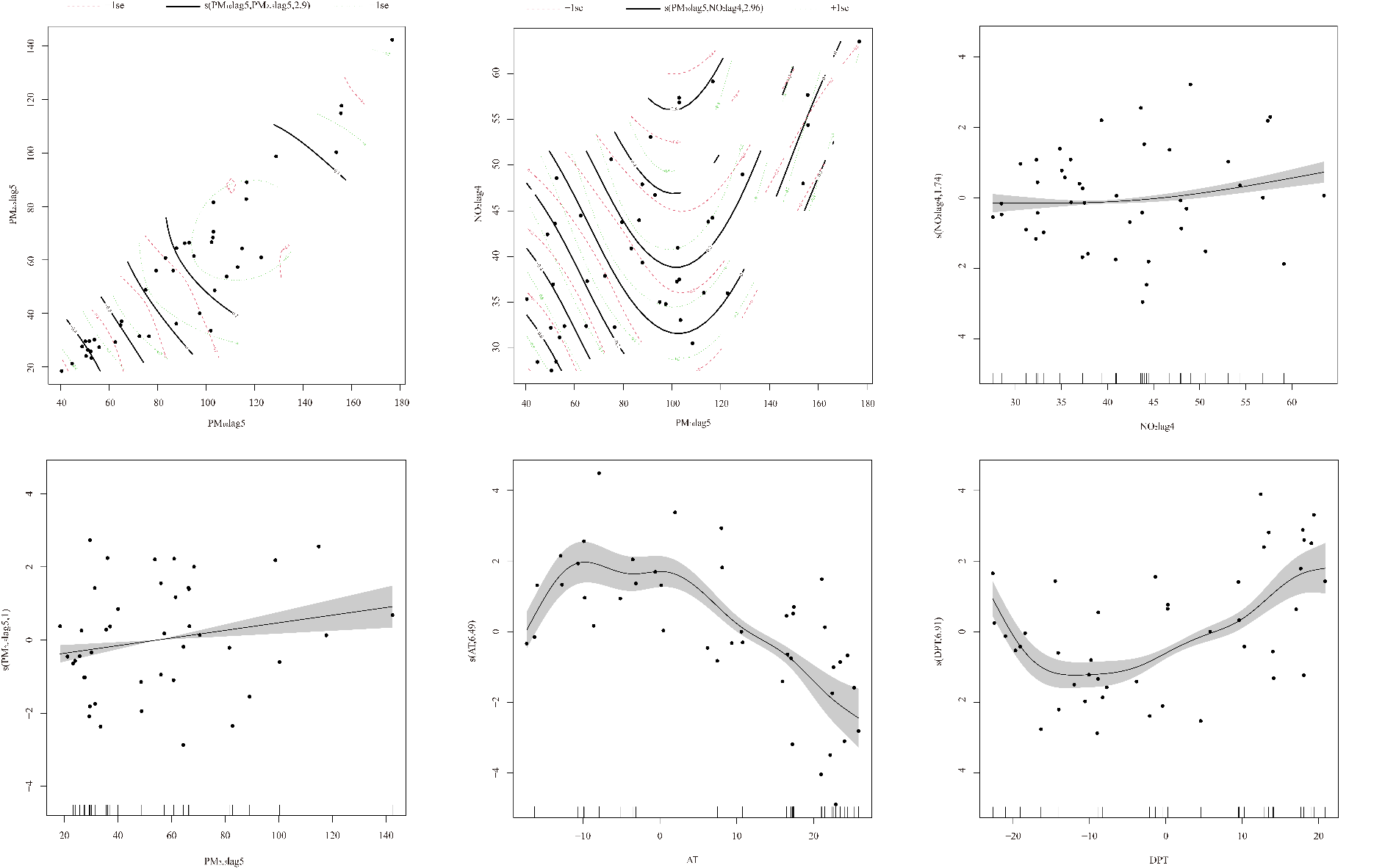


**Table S1** The estimated coefficients for least absolute shrinkage and selection operator (LASSO) regression among factors with influenza.

| Indicators | X±SD | Coefficients |
| --- | --- | --- |
| AT | 6.79±13.77 | -10.83 |
| DPT | -1.34±13.73 | -15.26 |
| SLP | 1011.34±18.48 | -0.69 |
| WD | 19.10±1.91 | - |
| WSR | 2.52±2.07 | - |
| AQI | 79.58±31.01 | - |
| CO | 0.96±0.25 | - |
| NO_2_ | 41.81±9.64 | -1.91 |
| O_3_ | 55.55±21.54 | - |
| PM_10_ | 86.15±33.66 | -2.56 |
| PM_2.5_ | 52.25±29.00 | -0.38 |
| SO_2_ | 32.93±30.99 | - |
| Model parameters | λ=28.3327 CV=5 | |
